# A Review on Perception of Binding Kinetics in Affinity Biosensors: Challenges and Opportunities

**DOI:** 10.1021/acsomega.4c10040

**Published:** 2025-01-27

**Authors:** Benjamin McCann, Brandon Tipper, Sepeedeh Shahbeigi, Morteza Soleimani, Masoud Jabbari, Mohammad Nasr Esfahani

**Affiliations:** ‡ School of Physics, Engineering and Technology, University of York, York YO10 5DD, U.K.; ¶ Department of Computer Science, University of York, York YO10 5DD, U.K.; § WMG, University of Warwick, Coventry CV4 7AL, U.K.; ∥ School of Mechanical Engineering, University of Leeds, Leeds LS2 9JT, U.K.

## Abstract

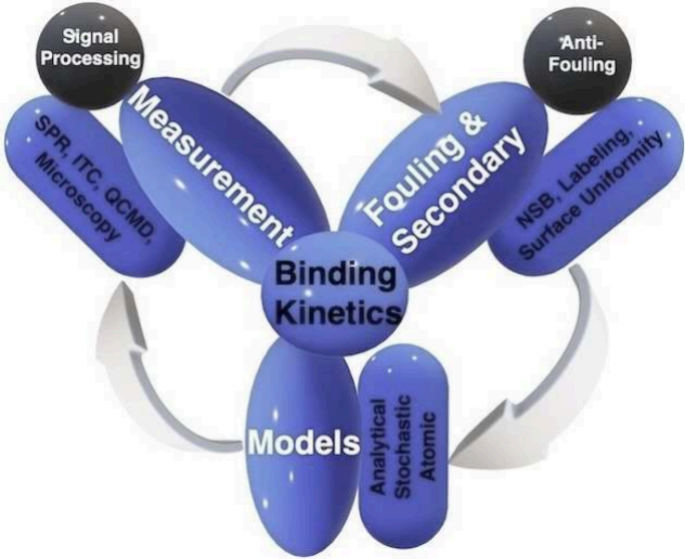

There are challenges associated with design and development of affinity biosensors due to the complicated multiphysics nature of the system. Understanding the binding interaction between target molecules and immobilized receptors and its kinetics is a crucial step to develop robust and reliable biosensor technologies. Evaluation of binding kinetics in biosensors becomes more important and challenging for clinical samples with a complex matrix. Despite drastic advancements in biosensor technologies, having a practical perception of the binding kinetics has remained a critical bottleneck due to limited fundamental understanding. This Review aims to provide a comprehensive discussion on concepts and advances developed so far for the perception of binding kinetics in affinity biosensors. Here, modeling approaches and measurement techniques are presented to characterize the binding interactions in biosensor technologies, while the effect of fouling and secondary factors in the binding interactions will be discussed in the concept of kinetics. This Review will investigate the existing research gaps and potential opportunities in the perception of binding kinetics and challenges to develop robust and reliable biosensors.

## 1 Introduction

Biosensors have been studied for several decades with extensive applications for clinical treatment, biomedical, pharmaceutical, and healthcare purposes. They are analytical devices incorporating biological elements in conjunction with physicochemical transducers to measure information about specific biological reactions or changes. Biosensors consist of four main components (or steps for detection), including (*i*) medium as a platform to transfer considered biological samples to a transducer, (*ii*) functionalized surface with immobilized bioreceptors to adsorb biological samples, (*iii*) transducer to measure biological reactions and (*iv*) computation to process information and transmit it into a simple and easy-to-use format. There have been extensive studies on these steps and components to offer opportunities and highlighting challenges to develop laboratory-based technologies and industrial products, a few of which are, progress of technologies,^1,2^ novel wearable devices,^3,4^ and innovative designing approaches.^5,6^ While there has been great advancement in biosensor components through novel detection strategies, biomarkers, modeling techniques, and applications, the of pace of progress has been different for such a complex system, leading to various gaps between laboratory-based devices and the requirement for on-the-spot sample analysis.^7,8^ The very rapid advance in detection technologies with unique features, such as high sensitivity, selectivity, and fast responses, left functionalized surfaces and biomolecular interactions behind to remain as new research questions for scientists. In this regard, several areas can be specified, including nonspecific molecules (as unknown molecules in clinical samples interacting with the functionalized surfaces), uniformity of receptors (their orientations and activities with target biomolecules), and the binding interaction between receptors and analytes. Some recent reviews have shed light on nonspecific molecules and characteristics of receptors, known as fouling in biosensors,^7,9−11^ while there has been limited attention paid to the binding interactions and kinetics, their roles in the biosensor performance, and challenges associated with existing assumptions made for the technology development.

This Review looks into the binding kinetics in affinity biosensors through presenting the existing literature developed so far to provide a better understanding of the biomolecular interactions, highlighting existing challenges and suggesting potential opportunities for future research studies. First, the fundamental thermodynamics of binding will be presented with a discussion on important phenomena during the affinity interactions. This will be followed by a review of the mathematical approaches developed to model the binding kinetics of biomolecules, mainly focusing on biosensors. After that, the effect of fouling and secondary factors (i.e., nonspecific binding and testing medium) on the binding interaction will be presented by evaluating their impacts on the binding kinetics. Then, well-known and widely used technologies to measure the binding kinetics will be discussed by highlighting their strengths and limitations. This Review will be concluded by discussing existing challenges in measurement and modeling approaches and potential opportunities to improve our perception of the binding kinetics for step changes to transform affinity biosensors for point-of-care applications.

## 2 Thermodynamics of Binding

**Figure 1:**
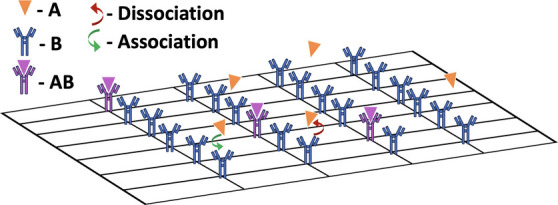
Schematic representation of the interaction of analyte molecules (A) with immoblized receptors (B) in a biosensor to create a complex (AB) through one-to-one association and dissociation. In this schematic, free analyte molecules and receptors are indicated as orange diamonds and blue Y-shapes, respectively. The same representation is used in other schematics for consistency.

The existence of bioreceptors as probes to create an interaction between analyte and transducer causes complexity in the detection process. Such a system can be described through two approaches. The first approach is to look into the interaction between analytes and bioreceptors and analyze the system from the perspective of molecular perception. The second approach is to study the energies in the system, such as energy barriers and binding energy leading to binding interactions. Hence, thermodynamics plays an integral role in defining the characteristics of biosensors. The binding interactions in biosensors are a complex system with many factors affecting the kinetics. This leads to difficulty in developing accurate sensors, where both the receptor and transducer components require thorough analysis before applications in a point-of-care (POC) device. In the following, a breakdown of different types of thermodynamic considerations and their influence on specific sensors will be discussed. It is important to make distinctions between the different types of energy present in the system, which defines various theories to analyze the binding interactions in a biosensor. Before discussing the thermodynamics of biomolecular interaction, first let us consider the binding interactions between analytes (A) and receptors (B) to form a complex (AB) (Figure 1) in a reversible reaction as
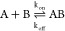
1

In this interaction, {A} represents an analyte moving freely in the solvent, and {B} is the binding site, known as the receptor, immobilized on the surface of the sensor. Finally, {AB} represents the analyte-receptor complex on the surface. The reaction being reversible means that analytes {A} can associate, with an adsorption rate, known as association *k*_*on*_, and dissociate from receptors {B}, forming and unforming analyte-receptor complexes {AB}, with a desorption rate, known as dissociation *k*_*off*_, respectively. This reaction is only approximated at equilibrium, where the system reaches a steady-state thermodynamic condition. Hence, there will be association and dissociation rates for such an interaction that keeps the amount of complex {AB} on the sensor surface constant. This binding interaction presented in Figure 1 will be used across this Review for the consistency of discussion. In the following, those parameters will be described with a discussion on their relations to the binding kinetics.

### 2.1 Gibbs and Artificial Energy Functions

**Figure 2:**
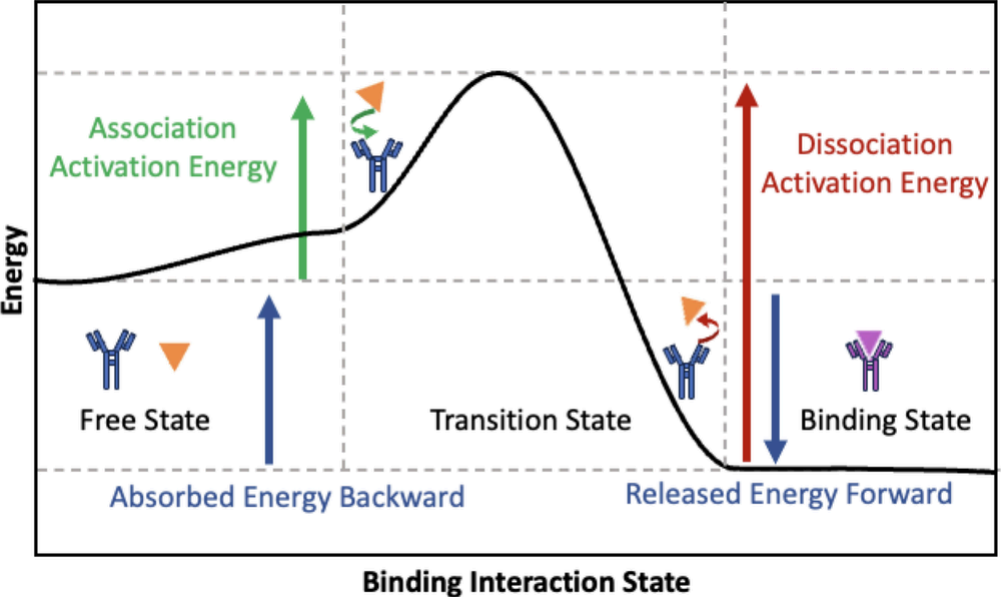
Schematic representation of the interaction states between analyte and receptor as a function of thermodynamic energies.

To provide a general overview of the free energy in a system with no constraints on the rigidity of the freedom of the receptor, one can take a statistical approach to solve the thermodynamics analysis through considering an artificial energy function by considering the coordinates of reactants (analyte and receptors) and the transition from their initial state to the final state of them. The artificial energy function includes all the contributions from the stochastic formulas for kinetic and potential energies, which can change with respect to the intermediary steps of a reaction. In addition to the artificial energy function, the energies of solvation also need to be taken into account by considering the entropic energy change. Hence, work needs to be done to allow an analyte either to dissolve in solution or to condense on a surface.^12^ This helps to estimate the Gibbs free energy, which can be used to calculate the association constant. Although, when the Gibbs free energy is found, it can be directly related to the binding affinity constants. The rate of association is much higher for reactions with a high level of Gibbs free energy, while the rate of dissociation in turn is much lower. Figure 2 shows a free energy diagram for a reversible reaction, in which the system releases energy in the forward direction and absorbs energy in the backward direction. As the amount of energy released is directly related to the Gibbs free energy and the reaction coefficient, the forward reaction is favored (*k*_*on*_) and the backward reaction is unfavored and much less likely to occur (*k*_*off*_). The transition state for a reversible reaction expressed in eq 1 is complicated, and it is important to note the activation energies in both directions. For the forward reaction to occur, a molecule must have sufficient internal energy to overcome the association activation energy boundary, which is a combination of factors noted in the potential and entropy energy sections. The Eyring theory can be used to model kinetic parameters,^13^ where the rate of reaction in both directions increases with respect to temperature. This again follows with the theory that more molecules on both sides of the reaction would have sufficient energy to overcome the activation energy boundary.

### 2.2 Kinetic Energy and Degrees-of-Freedom

The amount of kinetic energy a molecule possesses is primarily based on two factors: the temperature of the molecule and the number of degrees-of-freedom it possesses. Compared to simple diatomic molecules, biomolecules used in typical sensing applications have more complicated structures, resulting in many more degrees-of-freedom based on the structure of the analyte and complex. For example, anti-IgG has an Y-shape structure with 6 degrees-of-freedom. Another problem in biosensing systems is the flow rate, as increasing the flow rate in a system increases the velocity of a reactant flowing over a sensor’s surface, increasing potential and Gibbs free energies. In theory, this should reduce the likelihood of a successful binding event based on the previous Gibbs equation, though in turn, it should increase the chance that a molecule would possess enough energy to overcome the activation energy, enhancing the number of events. This results in complicated dynamics, especially in multiple step reactions. Nevertheless, it can be assumed that increasing the kinetic energy by raising the flow rate has a similar effect to elevate temperature, where both *k*_*on*_ and *k*_*off*_ increase.^14^

### 2.3 Potential Energy and Chemical Potential

The amount of potential energy of a molecule is primarily based on two factors. The first factor is the chemical potential, which is based on which bonds are broken and formed throughout the entire reaction chain. If more energy is lost in breaking bonds in the reactants than there is energy released to form bonds in the products, the forward reaction is less favorable. The second factor is based on how the structures of the reactants and products align with the solution. If a molecule is polar and contains dipoles that align well with the dipoles of the solvent, then work needs to be done to remove the molecules from their place in the solution. When analyzing chemical potentials, each type of bond has its own corresponding energy (under given conditions), and as such, chemical potential energies can be calculated assuming a reaction (and all its intermediary steps) is well-known. The potential energy of a solution can also be determined based on the polarity of the reactant/solvent and their corresponding radii based on a partition function, which describes the statistical properties of a system at thermodynamic equilibrium. Molecules containing strong dipoles in a polar solvent align in solution, with a prime example being how the negative backbone of DNA aligns with the positive hydrogen bond dipoles present in water. This leads to a higher association activation energy required to break this alignment and form free analytes before the construction of the AB complex (Figure 2). Hence, according to the Gibbs free energy, the higher activation energy requirement (of polar molecules in polar solvents vs nonpolar solvents) leads to a lower association rate of the reaction, assuming the reaction pathway is not significantly impacted from the change in solvent.

### 2.4 Entropy

The entropy of a system is a measure of stochasticity, or rather how many different possible formations are available to the system, with a large number of possibilities resulting in a larger entropy. This means that gaseous systems have more entropy than liquid systems and that systems with multiple analytes have a higher entropy than single molecule systems (due to the various permutations of both analytes). Stochastic models consider the solvation of both the analytes in solution and their receptor separately, creating an interaction potential of mean force that is solely dependent on the internal coordinates of the ligand, integrated with respect to the radii of the receptor and analyte.^12^ A comprehensive stochastic formula to denote the shift in entropy can be found elsewhere,^12^ along with the full derivation. When the thermodynamics of the system are known, the next step is conversion of the energy change from the reaction into a readable signal. For instance, the isothermal titration calorimetry (ITC) method, which will be discussed later in the Measurement Techniques section, measures the temperature of a cell with reactants in reference to another cell that contains only the solvent. By converting the shift in temperature to the total amount of energy released in the reaction, including the specific heat capacity of the solvent, solvation energies, and pressure, the total energy shift and hence the total number of formed complexes can be measured over a period of time. This provides a binding graph that is accurate so long as cells are well insulated and the thermodynamics of the system are well understood, while the effect of fouling, secondary factors, and environment of measurement, discussed later, can cause a degree of uncertainty.

## 3 Binding Kinetic Models

Beside thermodynamics approaches, the binding kinetics can be modeled based on molecular interactions. This section outlines modeling approaches that have been developed to study the binding kinetics of molecules. An overarching aim of this section is to illustrate the processes of each method, their applications, and limitations. This context is vital within sensor design to achieve optimum sensitivity and limit of detection with major impacts within expanding the efficiency of biosensors in their varied applications such as health care diagnosis and monitoring of environmental^15^ and food contaminants.^16^ Modeling the binding kinetics is also important for the measurement of the binding affinity of biomolecules using developed techniques, discussed in section 5. This information is important within biosensor design and the field of drug discovery. Understanding the binding kinetics is key to optimizing the sensor performance and thus detection of specific biomolecules. Furthermore, it allows evaluation of the efficacy of a medicine, as an index on how effectively a biomolecule binds to a target biomarker for a disease treatment (i.e., monoclonal antibodies for cancer treatment). Further details on the influence of the binding kinetics of biomolecules for drug discovery can be found elsewhere.^17−19^ In the following, well-know mathematical approaches for modeling biomolecule binding interactions will be presented followed by their strengths and challenges. These models include Langmuir isotherm, fractal methods, statistical methods, and atomic simulations.

### 3.1 Langmuir Model

The Langmuir model, also known as the Langmuir isotherm, is originally an empirical equation used for analyzing gases, which has been extended for liquid mediums.^20^ This model is performed at isothermal conditions to align with the definition of an adsorption isotherm.^21^ The Langmuir model is extended to liquid medium for biosensor applications, where the adsorption is the binding of an adsorbate, an entity such as biomolecule analytes, to a surface such as immobilized bioreceptors. This model assumes a monolayer coverage of all available analytes to receptors homogeneously across the surface of the sensor.^22,23^ In a biosensor concept, this means that analytes bind uniformly to the immobilized bioreceptors (Figure 1) on the surface, assuming identical interactions and binding sites across the sensor. The Langmuir model considers no heterogeneity on the surface,^20^ nonspecific binding and statistical collision. These factors in biosensors will be discussed along with other proposed models later in this section.

In the Langmuir model, the binding events can be assumed as a reversible reaction (eq 1). The governing equation of the Langmuir model considers a constant rate for *k*_*on*_ and *k*_*off*_, to estimate the rate of binding as a function of analyte concentration ([A]) and complex concentration ([AB]) at equilibrium. Furthermore, the Langmuir model assumes homogeneous surfaces and no interaction between neighboring molecules during the binding process.^22,24^ Furthermore, this model considers all sites at the same energy level,^25^ assuming the same energy level for desorption (unbinding) and adsorption (binding) processes.^24^ These assumptions make the model inaccurate for modeling all aspects of in vivo experiments and can result in frequent misuse of the model in the literature.^22^ The Langmuir kinetics can be formulated into the first-order or second-order binding kinetics based on the chemical reaction between analytes and receptors, where the ratio of available analyte to receptor capacity is a key determining the kinetics model.^26^ In the Langmuir model, the binding is assumed to have 100% probability in the case of an analyte reaching in contact with a receptor. Overall, the model represents the rate of binding, which can be used to estimate the binding kinetics parameters, association and dissociation constants, from empirical (experimental) data obtained from measurement techniques such as surface plasmon resonance (SPR) and quartz crystal microbalance (QCM). Those measurement techniques will be discussed in detail in subsequent sections.

Overall, the Langmuir model is classed as a simple model due to its limited parameters without considering any spatial, thermal, or other factors influencing the binding, e.g., the ionic strength of the buffer solution. Furthermore, there are some assumptions in the model that limit its application to predict the binding kinetics for certain systems. For example, this model assumes a high concentration of analytes so that it represents a homogeneous system on a macroscale. A recent study exhibits the failure of the Langmuir model for very small concentrations of receptors or analytes, where statistical approaches are required to capture the binding affinity of biomolecules in a small scale and close systems.^27^ This can be extended into the original model for neglecting any microscopic effects taking place within the binding, such as hydrophobic interactions or intracellular forces (Table 1).

**Table 1:** Summary of the Features for Different Methods to Model Binding Kinetics in a Biosensor

	Models					
Features	Langmuir	Fractal Analysis	Markov Chain	Langiven	Agent-based	Atomic
Ease of implementation	*√*	X	X	*√*	X	X
Computational complexity	X	*√*	*√*	X	*√*	*√*
Model surface and distribution of receptors	X	*√*	*√*	X	*√*	*√*
Consider *k*_*on*_ and *k*_*off*_ constant	*√*	X	X	X	X	X
Model fouling and secondary factors	X	*√*	*√*	*√*	*√*	*√*
Implement stochastic nature of binding	X	X	*√*	*√*	*√*	X
Model environment of binding	X	X	*√*	*√*	*√*	*√*
Scalable for a biosensor size	*√*	*√*	*√*	X	X	X
Model time scale comparable to measurements	*√*	*√*	*√*	X	X	X

The prediction of the model is accurate enough for data^28^ generated by commercially developed technologies based on the SPR technique, i.e. BiACore,^29^ discussed further in Measurement Techniques, to represent the average kinetics in the bulk of reactions. This model has a good accuracy for determining the main features of a reaction quickly, especially for well understood binding complexes, while stringent sample preparation methods are required to achieve reproducible results.^30^ This model can easily be implement into biosensor design for rapid prototyping to achieve optimal conditions for binding.^31^ The next part will discuss a model that allows for the consideration of binding in low dimensional space around clusters, which is challenging to perceive using Langmuir models.

### 3.2 Fractal Analysis

Fractal analysis is used to model the binding kinetics by using fractals^32^ to consider the heterogeneity of surfaces, which is neglected in the Langmuir models. It is noted that the fractal analysis has applications beyond the binding kinetics.^33^ Fractal analysis considers specific scenarios for the binding between target molecules and immobilized receptors on the sensor surface, where the Langmuir models are insufficient for modeling the binding interactions. This was first discussed by Kopelman in 1986,^34^ indicating deficiencies of the Langmuir isotherm to predict the binding limited to restricted space in clusters or by walls, phase boundaries created in heterogeneous biological systems. The surface of the biosensor is another reason creating heterogeneity in the binding between analyte and receptors with a considerable impact on the binding rate. In the case of a heterogeneous surface (known as deformities of crystal structure on the surface of materials), the association and dissociation rates are found to be time-dependent, where the fractal-like analysis can be employed to model such heterogeneity on the surface.^32,34−36^ In this model, the time-dependent binding kinetics parameters are referred to as rate coefficients. The reaction during fractal-like kinetics can not be approximated with the Langmuir model, as the reaction is disordered with irregular values of the kinetic parameters.

**Figure 3:**
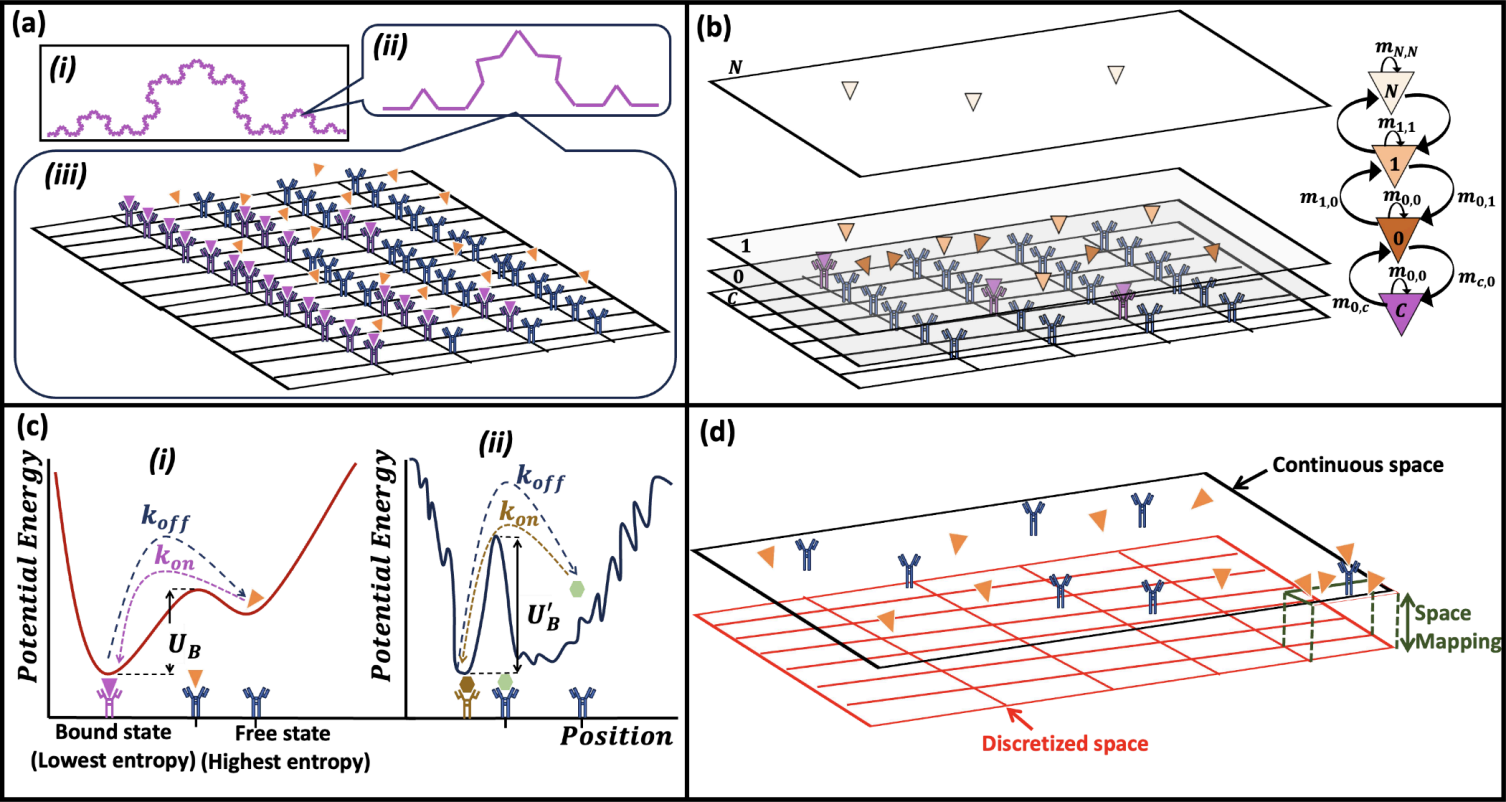
Schematic of (a) fractal-like binding kinetics representing (i)–(ii) clusters showing the binding on the surface of a sensor as fractal patterns, where the purple color shows the formation of an analyte-receptor complex, with (iii) an inset of the microscopic view of the surface and binding interactions, (b) a state transition diagram in the Markov chain model of an affinity biosensor, where each state corresponds to different coordinates, with state-c and state-0 presenting the binding state and interaction state, respectively, (c) the Langevin method demonstrating the binding of the (i) target molecule and (ii) nonspecific molecule, based on the change of the potential energy for a one-dimensional model along the distance between the molecule and receptor, and (d) a hybrid ABM with different discretized grids monitoring and influencing the behavior of agents.

In the low dimensional space of fractal analysis, analytes move around their original positions due to the compact space. In such an analysis, the reaction rate reduces overtime.^35^ In a diffusion limited system, the distribution becomes less random over time, leading to analytes likely revisiting their original positions in low dimension fractal-like reactions. This is in contrast to a classical homogeneous system with a uniform random distribution of analyte throughout the reaction. Therefore, as the analyte position is biased around these initial points in low dimensional spaces over time, it appears that the reaction rate plateaus due to uniform diffusion of analytes around their contained space. Hence, analytes are unable to discover receptors far from them, unlike the case of homogeneous higher dimensions (i.e., the Langmuir model in 3D). Figure 3(a) shows a schematic of a heterogeneous binding with clusters of binding complex across the surface of the sensor. The microscopic view of such fractal-link binding is presented in an inset (Figure 3(a)-(iii)).

The binding kinetics are perfectly described through using this model for both association and dissociation rates.^32^ An interesting result of this study is that the adsorption rate is sensitive to the level of heterogeneity of the sensor. The close binding between analyte and receptor in a diffusion-controlled system is the event defined as low dimensional. A specific example is mentioned previously^37^ through analyzing the kinetics between antibody antirabbit IgG and bovine serum albumin (BSA) measured in another study.^38^ They discuss the deficiency of using the first-order Langmuir model with constant binding kinetic parameters to fit the experimental measurements. Experimental measurements can be described by a diffusion-limited irreversible reaction, as their rate of association diminished over time.^38^ Fractal analysis was employed successfully to model experimental observations,^37^ where a weak dependence on the analyte concentration and surface disorder is reported. A high fractal dimension is linked to the heterogeneity and roughness of the sensor surface. Therefore, this fractal analysis outlines the disorder of processes on the biosensor surfaces.^37^ It should be noted that the fractal analysis can include any nonspecific binding (NSB) with increase of the fractal dimensions and the heterogeneity of the surface.^32^ Later, nonspecific molecules and secondary factors will be discussed in more detail with their impacts on the binding kinetics.

### 3.3 Statistical Models

Statistical models include microscopic processes with the probability of them occurring during the binding interactions between analytes and receptors. In these models, a time-dependent view into the binding kinetics is critical. The nature of binding is inherently probabilistic, where the binding occurs at a certain activation energy based on the thermodynamics of the system. These fluctuations of energy are random and can be described by probability functions, which determine whether a threshold potential is met. This threshold potential, for example, describes the required potential energy of an analyte to undergo conformational change or the energy required for binding to occur between analytes and receptors.^39^ This subsection will focus on the use of statistical approaches to model microscopic events influencing binding kinetics in biosensors. In the following, Markov models, Langevin methods, and agent-based approaches, as the main statistical methods, will be discussed with their applications on the perception of binding kinetics.

#### 3.3.1 Markov Chain

The Markov model is a probabilistic technique used to model dynamic systems.^40^ A Markov process is a sequence of random elements, where any next sequence element is predicted based on the previous state, irrespective of its sequence history before the last state.^41^ This model can be employed to describe the probability of transition from coordinate *i* to *j* at each time step presented in Figure 3(b), denoted by *m*_*ij*_. This model can estimate the signal-to-noise ratio of a biosensor.^42,43^ An advantage of such a mathematical model is the capability to include other time-dependent probabilistic behaviors influencing the binding kinetics in biosensors. This model can be used to represent various biosensor platforms such as electrochemical^43^ and ion-sensitive field effect transistor devices.^42^ Besides all the positive aspects, one of the main disadvantages of the Markov model is the systematic error introduced into calculations by discretizing the modeled space. This can be linked to the local behavior occurring in a continuous space. Boundaries define a discretized domain^44^ leading to interruption of a continuous space. The interruption of a continuous space means that there is no representation of local behavior on the boundaries of these grid lines, shown in Figure 3(b). Therefore, there is a loss of information around an analyte’s behavior, reducing the accuracy of the model, due to the lack of information to describe an analyte moving from one state to another. In practice, there is no discretization for the continuous space, except a different grid layer defined with a transfer matrix to convert the continuous domain into a discrete space.

The complexity of the Markov model is a challenge, especially in the construction of states. For systems with several variables for the process modeling, the computational complexity is intensive. For example, previous studies discuss requirements for further enhancement, such as describing dengue immunoglobulin positives and negatives to increase the size of the transition matrix. This is mimicked in other models for biosensors,^43^ where including many states to model a biosensor is difficult due to the size of different matrices including behaviors and their probabilities. Finally, the property of dynamics without a memory is found to be a disadvantage, where the transition between states is independent of previous states, leading to difficulty in deciphering trends from previous states. This reduces the capability of the Markov model to describe new phenomena. This is especially relevant to medical applications with disease progression being dependent on previous states.^45^ The Markov chain modeling technique has the ability to capture many processes occurring during the binding and including environmental factors which are missing from isotherm models. In this respect, the model is able to provide more insight into binding behavior through capturing stochastic microscopic details in time, such as nonspecific interactions. It is noted that the size of the model must be adjusted to allow for its efficacy, depending on the requirements and constraints on the optimization process.

#### 3.3.2 Langevin

The Langevin model is a stochastic equation that conforms to the Markov processes as described earlier.^46^ The Langevin dynamics model presents the response of a particle’s movement through considering microscopic forces such as thermal oscillations over time using stochastic differential equations.^47^ The original derivation of the Langevin equation describes the Brownian motion of particles by considering forces exerted on a particle.^48^ This model describes the binding as the change of an analyte’s potential energy based on its position in an energy profile of the binding process, illustrated in Figure 3(c). An unbound analyte in a free state (freely diffusing at a higher energy level) should overcome a potential barrier to bind. This corresponds to an initial increase in the potential energy for the creation of an analyte-receptor complex. The potential energy of the analyte is lower than its potential at the free state after the binding event. In this model, *k*_*on*_ describes the rate at which particles overcome this barrier for binding, whereas *k*_*off*_ represents the reverse of this process for the analyte to dissociate from the receptor and move back to its free state. The transition of an analyte between the free state and the bound state occurs from a time varying thermal noise term. This is because the stochastic thermal oscillation varies the potential energy of the particle to associate or dissociate by granting enough energy to create or break the complex molecule.^49^ The energy profile or funnel is smoother in specific binding (Figure 3(a)-(i)). This means a lower potential energy is required to overcome the potential barrier, leading to higher association rates. In the case of NSB (Figure 3(c)-(ii)), the potential energy profile is rough with a larger potential barrier hindering the binding events, while the thermal oscillations can still overcome the barrier, giving sufficient potential energy to the nonspecific molecules to form a binding to the receptor.^10^

The Langevin approach is applicable to modeling the energy profile of binding kinetics by including microscopic forces acting on analytes during their movement and the binding process. This higher resolution of microscopic behaviors is useful for generating more accurate and useful kinetic data. The inclusion of thermodynamic principles alongside Newtonian physics helps to give a more rigorous physical explanation of the processes that define the stochastic nature of binding kinetics. The main drawback in this type of modeling is computational complexity, especially in large systems with multiple forces acting on molecules. Therefore, this method is used for exploratory solutions of complex behaviors in biosensors such as nonspecific binding and electrochemical forces. In this regard, previous studies propose the use of an external force to control the binding kinetics in the context of dynamic single molecule sensors. The force modulates association and dissociation rates, *k*_*on*_ and *k*_*off*_, through changing the height of the potential barrier required to overcome the binding process.^48^ Such techniques can be used to overcome issues associated with nonspecific molecules in biosensors. In these techniques, *k*_*off*_ for nonspecific molecules bound to the sensor can be increased to be higher than that for specific binding, leading to a faster dissociation of those none-target molecules. Hence, the lifetime of the analyte-receptor complexes from specific binding on average last longer than those from nonspecific, helping to improve the accuracy of the sensor toward specific binding.

#### 3.3.3 Agent-Based

Agent-based modeling (ABM) is a discrete stochastic model considering individual characteristics and behavior in space and time of an agent.^50,51^ ABM approaches take individual behaviors into account by modeling each agent with a set of rules. These rules are dependent on the application such as modeling the Brownian motion of particles. The ability to model with rules makes them adaptable to different purposes. In the context of the binding kinetics and biosensors, ABM can be used to abstract any characteristic of individual entities to replicate the system behavior,^52^ which is challenging to model using other approaches discussed so far considering the biosensor as a unit, such as the Langmuir isotherms. A very high level of detail to consider in ABM provides an opportunity for implementation of various behaviors in biomolecular interactions, such as van der Waals forces, Lennard-Jones potential, and hydrophobic and long-range coulombs interactions. In this respect, treating agents in a spatial environment allows for the consideration of collisions between analytes,^53^ which has been neglected from models such as the Langevin equation. Another benefit of the ABM approaches is capability to model emergent behaviors,^50,52,54^ where a system behaves based on its set of fundamental rules without prior information about its surroundings. ABM has potential to explain certain elements of the binding kinetics, where other modeling techniques are unable to explain these. This provides opportunities to better understand the binding kinetics and biosensor performance prior to use of clinical samples for the development of point-of-care technologies.

It should be noted that some rules and parameters governing the agents are based on empirical evidence, where rules are applied based on probability functions at each time step according to analytical equations. The binding event in ABM occurs based on such probability functions. These empirical parameters used in ABMs help to explain the complexity of biomolecular interactions in a biosensor, such as fouling. Furthermore, they can also be used to validate empirical observations.^54^ Hybrid ABM approaches are widely used to model agents-based functions using multiple layers, representing different scales and further rules affecting the agent behavior. It is important to note that microscale behavior is handled with ABM and continuous modeling of macroscale behaviors is usually approximated using partial differential equations, which feed information to influence the microscale model.^55^ An example is demonstrated in Figure 3(d) with a continuous space (the agent containing layers) representing the microscale behavior of analytes and receptors moving around in a continuous space. These rules that govern at macroscale or microscale can be represented as discretized spaces using layers defined with lattices and meshes, which are used to map the continuous space.

Generally, the application of the ABM approach to model the binding kinetics can be considered for a model with the entire agents and process. They are scalable and adjustable to create a very realistic model depending on the considered computational complexity. They include microscopic interactions to model macroscopic behaviors while accounting for spatial and environmental interactions, which is challenging to replicate in differential equation models. Therefore, ABM approaches are attractive candidates for developing models to explore new theories explaining the binding interactions through the use of emergence to simulate known behaviors while also trialing new rules or behaviors. There are a few examples of ABM approaches used in biomolecular interactions.^53,56^ Furthermore, the ABM approach has also been developed to study tumor cell growth,^56^ where the cell interactions and phenotype developments are modeled in a continuous environment to understand tumor growth and the individual cell interactions with the environment. High computational demand is the main challenge to using ABM.

### 3.4 Atomic Models

The statistical models discussed previously consider analytes and receptors as individual particles and macromolecules. These models neglect any molecule conformation during binding interactions in biosensors. Atomic modeling approaches have been developed to study the system in full interactions between atoms through considering force fields to measure the physical properties of biomolecules such as conformational structures and binding mechanisms. Molecular dynamics (MD) simulations are the most well-known approach to modeling the binding kinetics at atomic scale. MD models take into account all atoms and forces at each time step, mainly employing Newtonian physics of particles, including van der Waals forces, electric charges between atoms, intermolecular bonds, hydrophilic and hydrophobic regions, and conformational and physical structures.^57−59^ Those simulation approaches consider all atoms involved and are mainly limited to model nanostructures. Hence, MD simulations are known as computationally expensive techniques, especially compared to other modeling approaches discussed here. In this respect, there are methods to reduce the computational intensity such as neighboring list and coarse grain methods.^60^ Considering the time scale in MD simulations, it is difficult to understand whether a system has reached thermodynamic equilibrium in microsecond time scales.^61^

Atomic simulations are extended to many applications especially in biomolecular analysis, e.g., modeling antiviral drugs to the capsid of rhinovirus^62^ and protein dynamics. There is great interest in using atomic simulations to understand the movement and conformation of proteins as well as their interactions.^62^ The structure of proteins is dynamic, causing challenges to understand their behavior. However, MD simulations are helpful in modeling complex phenomena driven by the dynamic nature of biomolecules. For example, MD simulations can be employed to determine the role of solvents at temperatures below the glass-transition temperature (the temperature at which a polymer changes from rigid glass state to flexible).^63^ A great impact of temperature on the amplitude of carbonmonoxy myoglobin protein oscillations has been reported, while this effect is found to be negligible at low temperature. Currently, this behavior necessitates rigorous techniques to observe experimentally, while the activity of proteins at different temperature ranges can be predicted using atomic simulations.^62^ A common implementation of MD simulations is protein docking. This is applied to understanding the kinetics of potential drug candidates through investigating the bind response of proteins to their targets for long residence times as an indication of the affinity of a drug target.^64^ MD models take into account thermodynamic contributions and the conformational structure. Despite the great potential to model the binding response, it is impractical to model dissociation events using MD simulations to estimate the kinetic parameters. That can be linked to the longer interaction time compared to the MD simulation time scale.^65^ Therefore, the incorporation of other models with MD simulations allows for the comparison of experimental data. For instance, previous studies use a two state model representing a reaction between an enzyme and an inhibitor based on the calculation of the mean first passage time.^66^

So far, we focused on approaches modeling the binding interaction between analyte and receptors in biosensors. In those models, we mainly assume that analyte and receptors are two main components of the interaction without any additional factors impacting the binding interaction such as nontarget molecules, known as NSB, fouling, and external forces in biosensors. There are certain features among those models that are listed in Table 1, representing opportunities and challenges to employ them for predicting the binding interactions in a biosensor. The next section briefly reviews any secondary factors (including fouling) influencing the binding kinetics in biosensors.

## 4 Fouling and Secondary Factors

**Figure 4:**
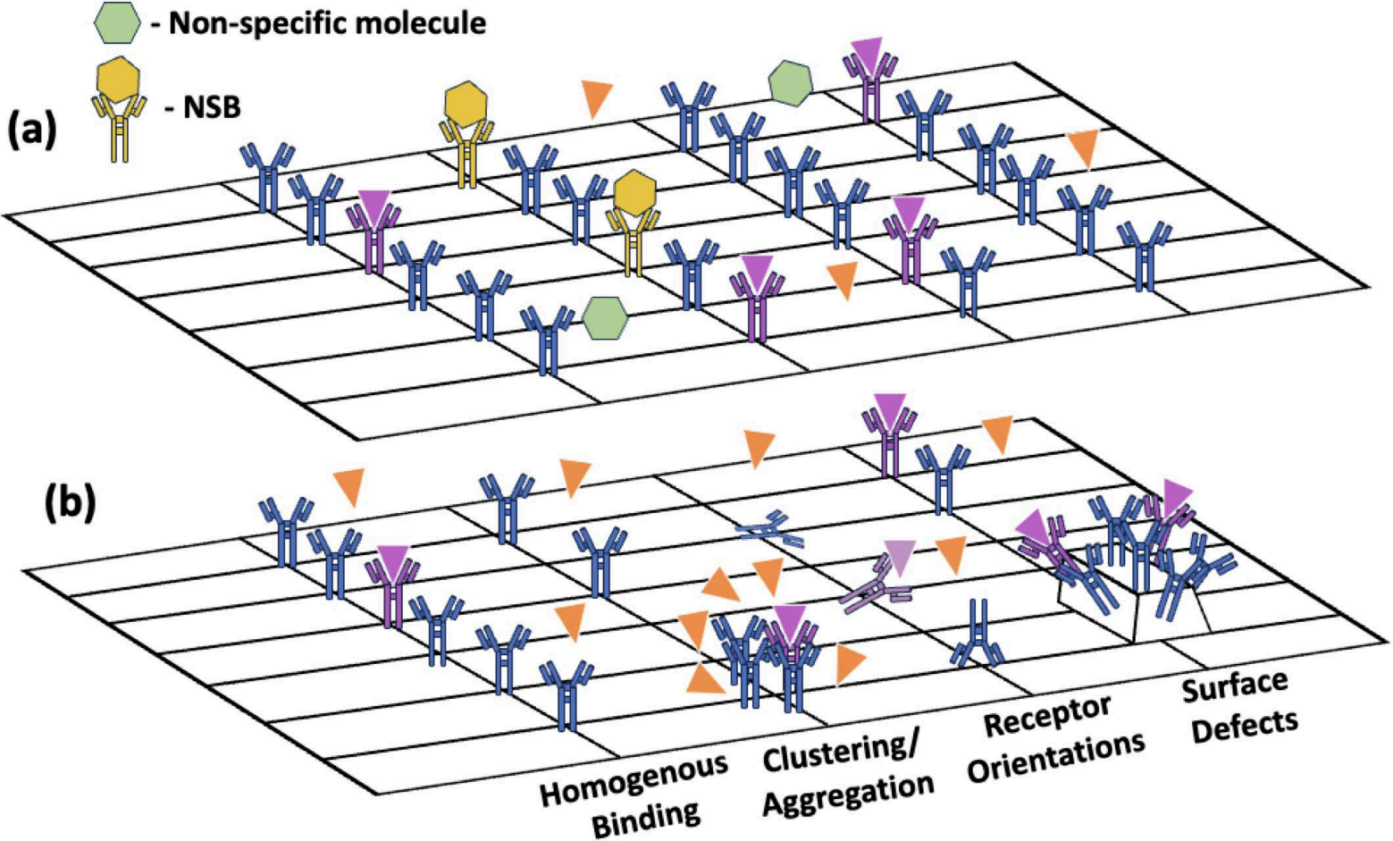
Schematic of (a) nonspecific molecules and NSB interfering target molecule binding interactions and (b) the receptor distribution and its impact on the binding, including homogeneous binding, clustering/aggregation of receptors, various receptor orientations (heads-on, side-on (with incomplete binding in faded purple color), and flat-on), and surface defects with nonuniform receptor distribution.

An ideal scenario for a biosensor is to have the target analyte and receptors as the sole components for interactions. Such an ideal system can be achieved in the laboratory through using stringent techniques, while there are various degrees of complexity, considered as fouling and secondary factors, in clinical samples such as nonspecific molecules interacting with receptors and sensors, distribution and orientation of receptors, collision and interaction with the sample matrix (i.e., serum), and analyte stability. This causes several issues in detection. First, they cause additional noise in the signal, as false-positive and false-negative signals. Moreover, interference of these factors leads to challenges to predicting the binding kinetics of the target analyte accurately. A schematic of those interfering factors is shown in Figure 4. This section will study the effect of fouling and secondary factors on the binding interactions and outline the methods used to optimize and reduce them. This review focuses on the binding kinetics, while there are several in-depth reviews on these factors for further details.^10,11,67^

The binding of unwanted molecules causes an increase in the generated signal from the biosensor. This introduces false-positives in the sensor outputs, where the signal represents both the target of interest and nonspecific molecules. These false-positives are equivalent to noise in the sensor signal reducing the sensitivity, signal-to-noise ratio and limit of detection.^68^ The issue of NSB enhances with the change of analyte concentration, with a scale of *N*^2^, where *N* is the number of molecules in the sample.^10^ This scaling inevitably becomes a significant problem in systems with high concentrations. Such scaling issues lead to more challenges with unknown level of fouling and secondary factors in biosensors prior to use in clinical samples.^69^ For instance, the nature of NSB is unclear, causing challenges to eliminating its effect during the use of biosensors in clinical samples. In laboratory settings, samples contain only analytes of interest, whereas real clinical samples such as blood or saliva contain not only the analytes of interest but also other particulates creating NSB to the receptor or transducer surface, leading to a high level of uncertainty in biosensors for use in clinical applications.^10,68^ It also causes issues with drug discovery and delivery, as it reduces the drug efficacy through interrupting the binding to cell membranes.^10^

At the molecular level, NSB causes complicated problems through interactions with both analyte and receptor molecules. The association between nonspecific molecules and receptor sites increases via cooperative binding,^10^ where receptors with two active sites (bivalent) have an analyte bound to one site. This binding of an analyte molecule to one active site of a bivalent receptor can change the affinity of the other binding sites.^70,71^ In this regard, a molecular binding (specific or nonspecific binding) to a bivalent receptor modifies the thermodynamics of the complex formation for other binding sites as a result of cooperative binding, which can enhance the formation of NSB compared to SB in biosensors.^10,70^ It should be noted that cooperative binding can provide a benefit to specific molecules through reducing the entropy for complex formation. Beside NSB, another behavior in biosensors is molecular crowding, also known as fouling, where the biosensor surface is internally crowded with many entities. Such crowding behavior can change the biomolecular (i.e., protein) behavior.^10^ A high concentration of these biomolecules leads to the excluded volume effect.^10,72^ The excluded volume effect refers to the space taken by one molecule as being unusable by another molecule, reducing the availability of the sensor volume.^72^ This leads to crowding around the sensor surface followed by a reduction of reaction rates, where biomolecules have challenges to moving freely around to bind with receptors, as other molecules cause collisions and block the path.^10^

The issue of fouling and NSB is generally inevitable, requiring certain methods to mitigate its effects.^68^ In this regard, the impact of NSB can be reduced with various techniques during the sensing process, for example, by placing a filter layer, physically or using a continuous-flow diffusion layer, between the transducer and receptor to inhibit molecules other than the target analyte from reaching the surface.^68,73^ A physical layer, also called a coating or nonfouling layer, is used to prepare the sensor surface to reduce NSB by covering surface defects of the sensor to prevent exposed areas from being available for NSB.^74,75^ Despite promising results, these techniques are still unable to provide the full requirements needed for clinical samples. Furthermore, antifouling methods are time-consuming and costly, with the possibility of causing damage to the sensor surface.^7,76^ There is also a technique called referencing that is frequently used to mitigate NSB effects on the signal outputs. The process involves using more than one channel, where NSB measurement is done separately to the specific binding, in a reference channel. It involves multiple measurements, and the output signal from the reference channel is then subtracted from the main signal, leaving the representation of only specific bindings in the signal. However, problems arise from the repeated measurements, as this increases the limit of detection.^10^ Other ideas include functionalizing different surfaces and using them as references for the signal. Further details of the surface modification methods can found elsewhere.^10^ Formation of a flow layer has been proposed to mitigate NSB challenges using laminar flow to create a continuous-flow diffusion layer to separate nonspecific molecules. This is also termed a continuous-flow diffusion filter. This technique has been used for in vivo blood sensing, where it separates a layer of buffer fluid from the rest of the sample with the nonspecific molecules to improve sensor specificity and reduce its limit of detection.^77^ Recently, the incorporation of nanostructures in conjunction with the electrohydrodynamic-driven fluid flow proposes a novel technique to enhance molecular interactions and reduce NSB.^78,79^ External force fields also can be used as an active method to achieve a similar mechanism to reduce NSB effects.^68^ Dynamic tracking offers a similar approach to mitigate the NSB issues through monitoring elements of the reaction overtime.^80^ All information for the system regarding analyte dynamics is required to have access to the unbinding time for each molecule to distinguish nonspecific from specific bindings, where the false-positive can be reduced from the output signal.

Environment of measurement and detection technologies are other secondary factors affecting biomolecular interactions and binding kinetics. In this respect, labeling techniques, such as fluorescence and nanoparticles, are used to improve sensitivity and specificity, while they can change the binding interactions and kinetics.^81^ For example, there is a significant impact of fluorescent labeling on the binding kinetics of lectin-glycoprotein interactions compared to other measurement techniques without labels.^82^ This labeling effect is found to be negligible on the protein–ligand binding interactions.^83^ Labels can also alter the binding interactions through damaging biomolecules.^84−86^ Beside those detrimental effects, nanoparticle labels are used in multiple areas across medical fields such as diagnostics, screening, and drug delivery by enhancing the binding interactions.^87^ For instance, nanoparticle labeling was employed in conjunction with SPR techniques to increase the binding affinity by enhancing the association rates.^87^ Further to labeling, nanoparticles are used to encapsulate engineered supramolecules to improve efficiency, in vivo stability, and biocompatibility.^88^ Nanoparticles can reduce the binding affinity as well. For example, gold nanoparticles can increase the dissociation rate (*k*_*off*_), leading to a weaker binding affinity.^89^ Further to changing the binding kinetics, nanolabels can agglomerate, leading to NSB.^90^ This effect can be reduced through functionalizing the surface of nanoparticles. For example, a mix of carboxylate and octadecyl groups functionalizing the surface of particles can reduce agglomeration and nonspecific interactions, while such a method changes the chemistry of the surface. Despite their interruption in the binding interaction, NSB can reduce the toxicity of nanomaterials.^10^

The role of surface chemistry on the binding kinetics and sensor performance becomes important in electrochemical technologies, where surface roughness, receptor orientations and their distribution (illustrated in Figure 4) can impact the binding interactions.^11,67^ In addition to the surface, conformational properties of some biomolecules (i.e., DNA and RNA) can change the binding interaction. A substantial portion of biomolecules, such as proteins and DNA, can change their conformational structure during the binding process with an analyte. The change of protein conformation acts as the transducer of the sensor, known as molecular switch sensors.^10^ They perform these changes specifically to certain analytes to mitigate the effect of NSB.^10,68^ In this technology, DNA is the binding site changing its structure (conformation) after interaction with analyte, which is detectable through various measurement techniques such as microscopic and electrochemical technologies.^10,91^ This approach is unable to fully remove the issue of NSB. Using an electric field is another approach to improve the surface chemistry for uniform receptors to enhance sensitivity.^92^ Recent studies have highlighted the influence of mechanical stimuli on molecular interactions to develop a dynamic environment for complex biological measurements.^93^

In order to reduce fouling and secondary factors, various strategies have been proposed so far involving the change of surface chemistry and the environment of detection. While these approaches are effective to some extent, they add more complexity into the system with considerable impacts on the binding interactions and, hence, the sensor performance.^7,94^ In this regard, a critical evaluation is required to assess the influence of those fouling and secondary factors on the binding kinetics. Single molecule biosensors are suggested as a solution to address those challenges.^39,48^ Some statistical modeling approaches (discussed previously) can predict those secondary factors (i.e. collision and NSB),^42,43,48,53^ while there are limited studies attempting to characterize the effect of fouling and secondary factors on the binding kinetics and biosensor design. More mathematical models can support antifouling strategies for the development of biosensors for clinical samples.

## 5 Measurement Techniques

To have a successful design of a biosensor, one needs to have in-depth information about the binding kinetics. There are several mathematical approaches to model the binding kinetics, as discussed previously, to predict the binding rates, as the number of complexes over time. Hence, precise measurement techniques are required to characterize the binding interactions for a meticulous biosensor design. In the following, the most well-known and commercially available technologies to measure the binding kinetics will be discussed, including surface plasmon resonance (SPR), biolayer interferometry (BLI), isothermal titration calorimetry (ITC), and quartz crystal microbalance (QCM). This will be followed by discussing key techniques used to characterize biomolecular interaction profiles. By carefully selecting a measurement technique that fits the purpose, the binding properties can be leveraged to achieve a clear view of biomolecular interactions with functional surfaces.

### 5.1 Surface Plasmon Resonance (SPR)

**Figure 5:**
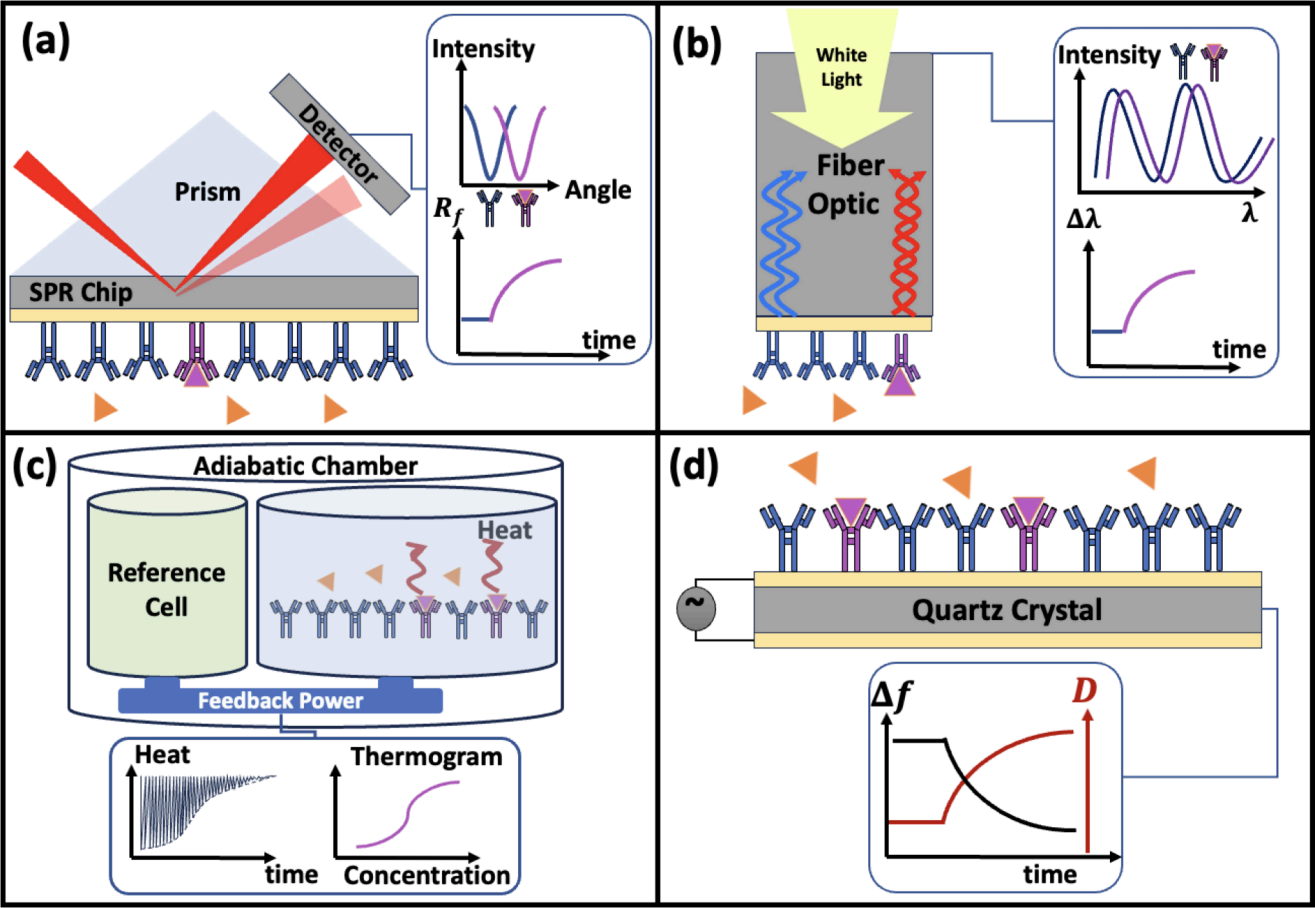
Schematic representation of (a) the SPR method to measure the binding interactions based on the refractive index of reflected light, (b) the BLI technique measuring the binding interactions based on the shift in the wavelength (λ) of reflected beams, (c) the ITC method to measure the binding interactions based on the heat exchange at the sample cell due to the binding interaction between analytes and receptors, and (d) the QCM-D method to measure the binding interactions based on the shift in the resonance frequency (Δ*f*) and dissipation (*D*) of quartz crystal as a result receptor-analyte complex formation.

The SPR technique is an optical technology using surface plasmon waves (electromagnetic waves) to measure the refractive index of a surface with the capability to detect biomolecular interactions (Figure 5(a)). This method measures the changes in the refractive index over an area with the evanescent wave.^95^ Therefore, it only detects binding events in a specific area on the functionalized surface.^96^ In SPR, the change of the refractive index can be measured as a response to a collection of mass from binding analyte to receptors as shown in Figure 5(a).^96−98^ SPR is one of the leading techniques to measure the binding affinity for biosensor design through offering high throughput and label free features,^96,99^ which is applicable for monitoring assays in real time, mutation detection, kinetic analysis, screening of drug candidates and identification of biomarkers for diseases such as cancer.^100^ The SPR method is sensitive to the optical thickness of the chip and its refractive index. Hence, this technique can be used to verify conformational changes. For instance, the protein conformation changes the refractive index in the SPR method.^100^ Regarding the limitations of this technique, there are challenges associated with data processing through using the Langmuir model to estimate the kinetic parameters,^28,101^ where the heterogeneity of receptors can cause a deviation from the first-order model. Furthermore, due to the small evanescent field domain, it is challenging to detect the binding kinetics of large molecules with a size greater than 10 μm.^96^ This technique has no capability to differentiate between specific and NSB, leading to false-positives during the measurement. It is noted that the specificity to distinguish binding profiles can be improved by using different materials for the chip. For example, a better evanescent field can be achieved through using silver embedded within gold for better detection capabilities for larger proteins.^102^

### 5.2 Biolayer Interferometry (BLI)

The BLI method is another optical technology based on the reflection of white light passing through optical fibers and interacting with a functionalized surface (Figure 5(b)). The binding interaction between analytes and receptors changes the wavelength of reflected beams, suitable for measuring binding kinetics. This technique offers nonfluidic sample delivery through moving the optical fiber across the solutions for a high throughput measurement in a low operation cost. This method can be easily integrated with other measurement technologies, such as microscale thermophoresis for measuring affinity in complex samples.^103^ Furthermore, thermodynamic parameters can be reliably measured by using the BLI method.^104^ Despite the great features in BLI, this method has a higher LOD compared with SPR leading to difficulty for measuring the binding affinity of small molecules.^105^ Similar to SPR, this technology is unable to recognize NSB, especially in weak interactions with *K*_*D*_ > 1 μM.

### 5.3 Isothermal Titration Calorimetry (ITC)

The ITC method is based on measuring the change of heat (calorimetry) in a reaction to predict the enthalpy, entropy, kinetics, and the stoichiometry between the two reactants using two cells, one as a cell of interest and another as a reference cell (Figure 5(c)). The amount of binding is determined by the change of temperature due to the binding interactions.^106^ The ITC technique offers accuracy comparable with SPR and BLI methods with capability to detect analyte concentrations in the order of nM to mM.^107^ This technique is widely used in drug discovery and pharmaceutical applications through predicting key thermodynamics parameters in biomolecular interactions.^108,109^ This method has fairly low throughput, leading to challenges to measure binding affinity. Moreover, the average time for each test is approximately 2–3 h with requirement of high concentrations of reactants.^107,108^ ITC is mainly used as a secondary exploratory technique beside other methods with high throughput to measure binding kinetics of unknown interactions.^110^

### 5.4 Quartz Crystal Microbalance (QCM)

The QCM technique is an acoustic wave technology used to measure binding affinity based on the shift in the resonance frequency of a quartz crystal resonator as a result of the mass adsorption the functionalized surface^111−114^ (Figure 5(d)). To have an accurate measurement, the mass must be rigidly bound to the surface without any slip. Hence, there should be no fluid friction between the surface and the buffer.^115,116^ A common variant of this technique known as QCM-D (standing for quartz crystal microbalance with dissipation) has been developed to address such challenges, where the dissipation of energy can be measured to determine the viscoelastic properties of absorbents.^117^ Measuring the viscoelastic properties can be employed to measure the orientation of the receptor as one of the main secondary factors (Figure 4) in the binding kinetics analysis.^11,118^ Furthermore, QCM-D has capabilities to detect protein conformation besides the interaction measurements,^119^ where monitoring the conformation effects gives an opportunity to identify NSB through using QCM-D along with the SPR technique.^120^ In this approach, SPR informs the density of the formed biolayer for the subsequent viscoelastic properties measurements using QCM-D, tested on estrogen receptor α-DNA complex. Further applications and use of QCM-D for the binding affinity can be found elsewhere.^121^

The main limitation of the QCM-D technology is a relatively low sensitivity compared to other methods,^122^ which is mainly driven from the properties of quartz crystals. This is mainly linked to the accuracy of estimating the binding complex from the shift in resonance frequency, which can lead to false-negatives during the measurements. In microfluidic biosensors, the flow rate must be optimized to further avoid such problems. For example, a high flow rate increases the noise on the signal, while a low flow rate can lead to false-positives on the signal due to proteins settling on the sensor surface without binding.^123^ The uniformity and crystal defects are other factors that influence the performance of this technology. Although QCM-D is a label free method, nanoparticles have been extensively employed in this technique to improve sensitivity and specificity.^122,124,125^ As discussed previously, considerations need to be taken into account for binding kinetics measurements.

So far, well-known and commercially available measurement technologies have been discussed with their features to analyze the binding kinetics, listed in Table 2. These techniques are mainly applicable for known biomolecular interactions with various limitations mainly on the affinity range and the size of biomolecules. Hence, several methods have been developed to address those challenges. In the following, these techniques will be discussed, including scanning tunneling microscopy (STM), surface-enhanced Raman scattering (SERS), nuclear magnetic resonance (NMR) spectroscopy, X-ray microscopy, and total internal reflection fluorescence (TIRF).

**Table 2:** Features of Well-Known and Commercially Available Techniques to Measure the Binding Kinetics of Biomolecules, Including Limit of Detection (LOD), Affinity Range (*K*_*D*_), and Their Strengths and Limitations

Methods	LOD (ng/cm^2^)	*K*_*D*_ (nM)	Strengths	Limitations
**SPR**	0.01	0.1–10^3^	Well-established in commercial level, high throughput, high level of repeatability, measure binding affinity and molecule conformation, label free	Dependent on the uniformity of receptors, limited to biomolecules smaller than 10 μm, not able to detect NSB
**BLI**	0.1	0.01–10^6^	Offering nonfluidic sample delivery, high throughput, low operation cost, measuring thermodynamics of interactions, suitable for high affinity interactions	Limited to large molecules, not able to detect NSB, poor reproducibility
**ITC**	-	1–10^5^	Predicting thermodynamics of interactions, highly sensitive, measuring unknown interactions, label free, no need to immobilization processes	Low throughput, high cost, large sample consumption
**QCM-D**	0.5	0.1–10^5^	Well-established in commercial level, high throughput, high level of repeatability, Measuring orientation of receptors, possibility to monitor molecule conformation and NSB, label free	Low sensitivity, accurate estimation of the number of complexes

### 5.5 Scanning Tunneling Microscopy (STM)

The STM technique is based on quantum tunneling to generate an image of the surface, allowing for understanding the structure of molecules as well as characteristics of the functionalized surface through controlling the movement of a molecule through rotation, conformation or translation.^126^ There are different approaches to employing STM for the binding kinetics measurements. One main approach is to functionalize the probe tip with a target molecule passed over the receptor,^127^ where the bond type and lifetime of the complex can be measured based on the binding response at a single molecule level.^128^ STM is able to measure the molecule transfer during the diffusion process and complex formation.^126^ A very low throughput and complexity of the control systems and high operational cost are the main limitations of the STM technique to measuring the binding kinetics.^129^

### 5.6 Surface-Enhanced Raman Scattering (SERS)

The SERS method relies on the change of the Raman spectrum based on the chemistry of the surface during the biomolecular interactions.^86,130−132^ Due to its noninvasive properties, this method is suitable to detect binding interactions in live samples such as cellular processes.^132,133^ SERS is able to distinguish the structure of binding suitable for NSB measurements,^131,134^ which makes it suitable for complex samples. Nanolabels are widely used with this method, which can cause challenges associated with fouling and secondary factors during the binding kinetics measurements.^130,135^ Challenges of reliable and reproducible measurements, difficulty to capture large molecules and operational costs are the main limitations of this technique.^136^

### 5.7 Nuclear Magnetic Resonance (NMR) Spectroscopy

The NMR technique detects the structure and chemical environment of molecules on the functionalized surface with capability to measure the binding interactions.^137,138^ NMR is able to measure weak to medium binding affinities with dissociation constants *K*_*D*_ from μM to mM, while this technique has difficulties measuring the binding kinetics with affinity less than 1 μM.^139^ NMR has a very high sensitivity to the chemical environment of atoms suitable for determination of NSB in complex samples, especially for protein–ligand interactions.^138^ Furthermore, this method has capability to measure the physical and conformational properties as well as thermodynamic properties as key aspects in the binding kinetics analysis.^10^ As a noninvasive method, NMR can be employed to measure the binding interactions in live samples. Despite a good throughput for rapid detection, NMR requires complicated processes for sample preparation.^138^ Moreover, this method relies heavily on labeling, especially for proteins, where secondary factors can impact the binding kinetic measurements.

### 5.8 X-ray Microscopy

X-ray microscopy is based on the beam diffraction of the functionalized surface to determine the structure of biomolecules for the binding interaction analysis. This method is used to measure the binding kinetics as well as conformation of receptors.^140^ X-ray microscopy can monitor the structure of a reaction over time through monitoring thermodynamic parameters.^141^ This method offers fairly high throughput detection, enabling the binding kinetic measurement of thousands of ligands with a target protein. Furthermore, NSB can be recognized through this technique,^142^ while the sample quality plays an important role on the accuracy of the measurement.^143^ This method is limited to crystalline molecules, where cryogenic measurements are needed for some biomolecular analysis with potential impacts on the binding response. The X-ray beam can also damage biomolecules under study with subsequent effects on the binding kinetics.^144^

### 5.9 Total Internal Reflection Fluorescence (TIRF)

TIRF microscopy is an optical technology based on the interaction of light with different media to create various refractive indexes.^145^ TIRF is widely used to measure the binding kinetics,^146−148^ while it relies on the labeling processes for the binding interaction analysis. In addition to the binding kinetics, the method can also determine conformational changes by analyzing the fluorescence signal.^146^ Such a specific detection offers the capability to measure NSB through using nanoparticles.^149^ TIRF offers a unique microscopic technique with high sensitivity and low photodamage without any limitation on the number of labels applicable for a wider range of biomolecules. Besides those advantages, TIRF has operational complexity in the labeling process and data analysis, where the impact of labeling on the binding kinetics needs to be considered, as discussed in the previous section.

In this section, most well-known techniques to analyze the binding interaction of biomolecules have been presented. These techniques are employed to measure the binding affinity and kinetics parameters for biosensor design.^31,150^ There are two main issues with such an approach. First, binding kinetics parameters are significantly dependent on sample preparation during the measurement. For instance, there is a high level of uncertainty on binding kinetics measurements using the SPR technique as a result of sample preparation.^30^ This can cause misleading design of affinity biosensors. The discrepancy between various techniques is another challenge to consider to employ the measured binding kinetics parameters for affinity biosensor design.^151^ Recognizing fouling and secondary factors, discussed in the previous section, from output signals in biosensors is a challenging process. In the following, well-known signal processing methods used for biosensors and measurement techniques will be presented.

### 5.10 Signal Processing

One of the effective methods to mitigate fouling and secondary factors and improve the signal-to-noise ratio is processing signals obtained from biosensors. In this regard, various signal processing methods have been developed where Fourier transform, wavelet transform, and traditional filtering methods such as Kalman filters are widely used for biosensor applications.

#### 5.10.1 Fourier Transform

The Fourier transform method enables the decomposition of complex signals into their constituent frequency components, unveiling underlying patterns and dynamics. By transforming signals from the time domain to the frequency domain, this method facilitates the identification of characteristic frequencies associated with biological interactions, allowing for the sensitive and specific detection of biomolecules in samples. This is especially useful in applications such as DNA microarrays and protein sequencing, where the frequency of certain markers can indicate the presence or absence of specific traits or conditions.^152^ One key step to analyze a signal from a biosensor is to transform data into biomolecular interactions, where signal processing can help for this purpose. For instance, Fourier transform is employed in spectroscopy devices to convert infrared spectral data into a molecular absorption and transmission spectrum, providing a distinctive molecular fingerprint and facilitating the identification and characterization of bacterial strains based on their unique molecular compositions. Fourier transform can decipher binding events and alterations to detect a myriad of biological entities.^153,154^ Despite its profound impact, the use of the Fourier transform method for biosensors is challenging. In this regard, Fourier transform-based methods can have poor performance for detecting analytes at exceedingly low concentrations, necessitating amplification mechanisms.^155^ Furthermore, the inherent mathematical complexity poses a barrier to the implementation of Fourier transform techniques, especially in portable biosensors.^156^ The interpretation of Fourier-transformed data demands expertise, particularly in complex biological matrices in clinical samples.

#### 5.10.2 Wavelet Transform

Wavelet transform has emerged as a versatile tool in signal processing, offering unique advantages in capturing both time and frequency information simultaneously. Unlike traditional Fourier-based methods, which provide a global view of signal frequencies, wavelet analysis allows for localized frequency analysis, making it particularly well-suited for analyzing nonstationary signals often encountered in biosensor applications.^157,158^ One of the key advantages of wavelet transforms in biosensor applications is their ability to denoise and baseline-correct signals. The linear decomposition of signals into different frequency bands allows for the effective removal of unwanted noise and background interference, which is crucial for improving the signal-to-noise ratio and accurate detection in biosensors. In this regard, the efficacy of wavelet-based adaptive denoising and baseline correction is demonstrated through decomposing the signal into various frequency components, allowing for the identification and removal of noise components while preserving the essential features of the signal to identifying biomarkers from human serum/plasma.^159^ Additionally, wavelet-based methods have been utilized for feature extraction and signal compression, facilitating efficient data processing and storage of biosensor systems. Unlike denoising and baseline correction, which primarily aim to improve signal quality, feature extraction involves identifying and extracting relevant information or characteristics from biosensor signals. In this regard, wavelet transforms are utilized to identify specific features or patterns within biosensor signals that correspond to analyte concentrations or other relevant parameters.^160^ This approach can help to enhance the sensitivity and specificity of the biosensor. More details of emerging applications of wavelets, including their utilization for signal compression in biosensor systems, can be found elsewhere.^161^

#### 5.10.3 Traditional Filtering

Traditional filtering techniques, such as Kalman filters and frequency filters, are commonly used in biosensor applications to ensure accurate and reliable measurements. The Kalman filter and its extended version, the Extended Kalman Filter, have found widespread applications in various domains, including biosensor technologies. These state-space estimation techniques have proven to be effective in tracking and predicting the behavior of complex systems, making them suitable for processing data from biosensors. The Kalman filter is a recursive algorithm that estimates the internal state of a dynamic system from a series of noisy measurements. It operates in two distinct phases: prediction and correction. The prediction phase uses the system’s state transition model to estimate the current state, while the correction phase adjusts this estimate based on the latest measurement. This allows the Kalman filter to provide reliable estimates of the system’s state, even in the presence of noise and uncertainty. An Extended Kalman Filtering Projection Method was introduced and applied to enhance the performance of optical biosensors by reducing the 3σ noise value.^162^ The Extended Kalman Filter was employed to refine the estimation of noise parameters, particularly the noise covariance matrix, thereby improving the accuracy of sensor measurements.

Frequency filters are utilized to selectively pass or attenuate specific frequency components of signals obtained from biosensors. By filtering out noise or unwanted signals, frequency filters enhance the accuracy and reliability of data interpretation. In biosensor applications, frequency filters are particularly useful for isolating and analyzing specific molecular interactions, enabling precise detection and quantification of target analytes amidst complex biological matrices. They play a crucial role in improving the sensitivity, specificity, and overall performance of biosensor systems. The application of a low-pass filter as a type of frequency filter played a crucial role in understanding the dynamic response limits of affinity-based sensors. By employing this filter in the frequency domain, the effects of diffusion, convection, and reaction on sensor performance can be isolated to better analyze the continuous sensing systems by filtering out high-frequency components such as rapid fluctuations in analyte concentration.^163^ Those filter methods use mainly the Langmuir isotherm through considering the binding interactions without implications of fouling and secondary factors, as discussed previous, while there are some studies incorporating band-pass filters with statistical binding kinetics to capture the noise driven from secondary factors, such as nonspecific bindings.^42,43^

Those filtering methods discussed so far are used to improve the accuracy of biosensors as a fundamental criterion in the evaluation of clinical and laboratory tests with complex samples to enable identification of the presence and level of a specific analyte. There is a trade-off between sensitivity, known as the ability to correctly detect true positives, and specificity, considered as the ability to correctly identify true negatives. Across this balance, there are the concepts of false-positives and false-negatives, which correspond to the test’s intrinsic statistical errors, known as type I and type II errors, respectively.^164^ False-positives, or type I errors, occur when a test incorrectly indicates the presence of an analyte in a blank sample. The presence of an NSB is an example of type I errors. False-negatives, or type II errors, arise when a test fails to detect an analyte that is present in the sample. Lack of proper functionalized surfaces and fouling are the potential source of type II errors. The statistical approach to controlling them typically involves setting a predefined level for errors, commonly at 5%. The chosen error thresholds have an impact on the limit of blank and the limit of detection, which are critical to establishing the test’s ability to differentiate between true negative and true positive results. Some examples of those errors have been discussed early, such as false-positives in QCM-D with low flow rates,^123^ NSB in detection of small molecules,^10^ and false-negative due to receptor clustering^11^

Minimizing these errors is thus crucial in the design and validation of diagnostic assays. For instance, increasing the number of replicate measurements or reducing noise in the system can lower both the standard deviation of blank samples and minimum level to detect analyte, thereby improving both the limit of blank and the limit of detection and ultimately enhancing diagnostic sensitivity. These steps are suitable for laboratory sample testing and preclinical development stages, while further statistical analysis and evaluation metrics are required for clinical applications.^164^ In addition to signal processing, several different approaches have been proposed to mitigate false-positive and false-negative errors during the measurement. For example, decreasing sensitivity of the biosensor outputs can significantly eliminate false-positives, where incorporation of a prescreen can further reduce true negatives during the measurement process.^165^ Using machine learning methods is another approach recently proposed to reduce the impact of false-positives and false-negatives in biosensors.^166^ Employing appropriate binding kinetics models and measurement approaches should be considered to explore those false errors in the signal processing steps. For instance, the Markov models, as discussed previously, are suitable to capture NSB and false-positives in noise,^42,43^ while the SERS technique can distinguish NSB in complex samples.^131^ Hence, having a proper evaluation of binding kinetics can help to design reliable biosensors, which will be discussed in the following section through presenting current breakthroughs to analyze binding kinetics in biosensors.

## 6 Challenges and Opportunities

**Figure 6:**
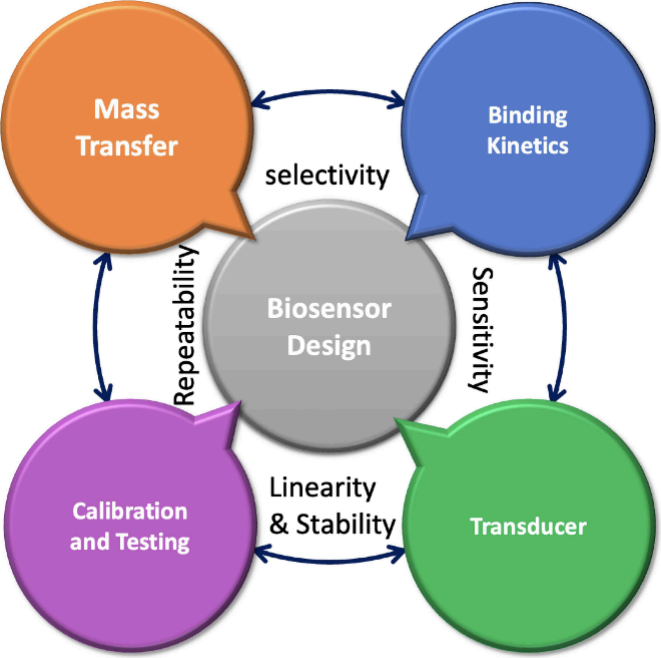
Schematic representation of key steps to design affinity biosensors, where binding kinetics is integrated with other aspects including biomolecule mass transfer through media, signal generation through sensor’s transducer and standard calibration curve and testing.

Understanding the binding kinetics is one of the main pillars to design affinity biosensors. Figure 6 demonstrates the main pillars to design a biosensor, including media for mass transfer, surface chemistry for binding, transducer to generate a readable signal, and testing for calibration curves, which determine the fundamental performance parameters, including selectivity, sensitivity, linearity, stability, and repeatability. In this design procedure, analyte mass transfer (convection- or diffusion-based physics) is integrated with the binding kinetic model, discussed earlier, to predict the binding response, where the binding kinetic parameters (*k*_*on*_ and *k*_*off*_) are measured using techniques such as SPR, QCMD, BLI and ITC, as discussed previously. Then the binding concentration can be linked to the transducer response based on the transaction mechanism of the biosensor technology. This will be compared to testing and calibration curves through appropriate signal processing, where the optimum design can be achieved through multiple iterations across these steps. Due to its simplicity, currently the Langmuir model is widely used in the binding response prediction,^31,150^ binding kinetic measurements^30^ and transducer signal processing.^162^

Besides all challenges associated with the Langmuir model discussed previously, binding kinetics is considered constant, assuming uniformity in the binding sites. This model development in biosensor design relies on a very high ratio of analyte to binding sites, where all binding interactions can reach the equilibrium state in a small period of time, leading to various problems.^22^ Such assumptions are beyond the Langmuir isotherm, where other binding kinetic models such as fractal analysis, Markov chain, and Langevin have the same considerations on equilibrium in the binding interactions. Besides these limitations, inappropriate procedures are other issues causing challenges in having accurate binding affinity measurements and biosensor design. A recent investigation has evaluated several studies in the literature on the binding affinity estimations, highlighting the importance of measurement procedures on the accuracy and reliability of outcomes.^167^ Hence, a framework is proposed to ensure the reliability of equilibrium approaches used to determine the binding affinity of biomolecules. This framework is based on providing sufficient time to achieve equilibrium and controlling the titration regimes. In this regard, varying incubation time and biomolecule concentration can help to ensure equilibrium in the binding regime during affinity interactions, where models can be employed accurately to determine binding kinetics as well as design biosensors. Using such a standard framework can provide opportunities to remove uncertainties associated with measurement procedures as well as to better understand limitations in current modeling approaches.

The use of equilibrium approaches to measure binding kinetics and design biosensors requires knowledge of the biomolecular interactions and their affinities. Furthermore, significant noise in a low concentration of analyte and recognizing a fully developed equilibrium state in a biosensor are some other challenges.^167^ In this regard, a recent approach has been proposed to determine the binding affinity based on the transient state before reaching the equilibrium condition.^168^ In this method, the rate of association with respect to time and input concentration is passed backward into a target estimation algorithm based on an inverse Langmuir calculation with a high pass filter. Further to maximizing signal-to-noise ratios, this technique offers biosensor design for continuous monitoring systems. This new approach to designing affinity biosensors can help with better incorporation of binding kinetics into signal processing without concerns to reach equilibrium. The application of such a method depends on the relationship between the concentration oscillations and transducer response.^163,168^ There are many opportunities to measure the binding kinetics of low affinity interactions, to eliminate the noise of NSB and design reliable biosensors for continuous measurements. In addition to pre-equilibrium techniques,^168^ controlling binding affinity through molecular switching is another approach to overcome challenges associated with the equilibrium methods. There are several recent proposals in this regard, where the binding kinetics (both association and dissociation rates) can be tuned in reversible interactions.^169−172^ These techniques provide several opportunities. First, the thermodynamics and kinetics of interactions can be decoupled for detection of specific binding. In this respect, the Langevin model works well to predict the change of thermodynamics in the binding interactions, where NSB can be distinguished.^48^ Detecting low affinity interactions is another opportunity to use molecular switching through enhancing binding kinetics. Besides fast and sensitive detection, these techniques provide opportunities for continuous measurements as well without concerns on sample preparation and measurement procedures.^169^ Developing high-speed switching is another application of using modulated binding affinity for modern electronic devices.^173^

The applicability of binding kinetics in different biological samples is crucial for advancing the design and utility of biosensors in diverse real-world settings. Binding kinetics directly influences biosensor performance in terms of sensitivity, specificity, and response time across various biological matrices. Understanding multivalent protein interactions is one example of the importance of binding kinetics to identify and develop novel pharmaceutical strategies.^174^ Precise targeting and imaging in vivo are other aspects on the significance of binding kinetics for dynamics monitoring of therapeutic processes.^175^ In simple biological samples such as buffer solutions or cell culture media, binding kinetics are relatively straightforward to measure and predict. There are many studies on exemplar biomolecular interactions in buffer solutions such as antibody–antigen, DNA, and Biotin–Avidin.^30,83,147^ These environments often lack complex interfering substances, allowing for the robust quantification of analyte-receptor interactions. However, in more complex biological samples, such as blood, saliva, or urine, binding kinetics become significantly influenced by secondary factors.^10,176^ The complexity of biological samples has an impact on the biosensor characteristics, such as reducing selectivity. These effects necessitate careful optimization of surface chemistry, such as the use of antifouling coatings and incorporation of referencing strategies.

The variability of binding kinetics across biological matrices highlights the need for robust sensor calibration and adaptation to specific sample types. Addressing these challenges involves integrating kinetic modeling with empirical measurements across different matrices. Advanced models that incorporate real-time environmental factors, such as viscosity and molecular crowding, can improve predictions and guide biosensor design. This enables the reliable application of binding kinetics for diagnostics and biomarker discovery across diverse biological samples.

## 7 Conclusions

The main purpose of this Review is to explain the concepts and advances of binding kinetics and its importance in design and development of affinity biosensors, which have been overlooked in the development of robust and reliable technologies for clinical samples with a complex matrix. Here, the fundamentals of binding interaction are presented through thermodynamics and energy concepts. This is then extended to the binding kinetics models proposed so far, where the strengths and limitations of those models are discussed for designing affinity biosensors. The implication of fouling and secondary factors (such as environment of measurement, nonspecific binding and sample medium) on the binding kinetics is then discussed as various noise sources in the biosensor responses. This Review is then concluded through the evaluation of experimental techniques to measure the binding kinetics of biomolecular interactions. The existing challenges and potential opportunities on the current perception of binding kinetics can be summarized in the following:

- Understanding the thermodynamics of binding in a biosensor provides an in-depth insight into the profile as well as statistical nature of binding, while there are limited theoretical and measurement studies. Current advanced technologies (such as STM, SERS and NMR) can offer high resolution measurements for the thermodynamics of binding, where the binding interactions can be looked at from fundamental principles. This can provide opportunities to distinguish the binding interaction profiles between analytes and receptors with different orientations and distributions.
- The Langmuir model is a simple approach to consider, especially in design and signal processing, while this model is unable to consider fouling and secondary factors as well as the statistical nature of binding for practical measurements and complex samples. In contrast, statistical approaches, such as Markov Chain, Langevin and Agent-based models, can offer opportunities to address those issues in biosensor design, while the challenge associated with their computational complexity and scale can be resolved through proposing multiscale models.
- It is too idealistic to have a universal model to design all biosensors, so mathematical models need to be developed to fit for purpose. One such purpose could be better understanding of the complexity of binding, where mathematical models can support various steps in biosensor design including strategies to immobilized bioreceptors, techniques to improve surface chemistry, and approaches to control fouling. This can lead to the improvement of reliability and repeatability of biosensors, especially for POC applications.
- There are various techniques, as presented in the last section, to measure the binding kinetics, while careful considerations are required in order to use those affinity binding kinetics parameters for the design of biosensors, especially with different technologies. As discussed in this Review, secondary factors can have a range of impacts on the binding kinetics based on the measurement technique. In this respect, a mathematical model can provide insights on how to transfer the binding kinetics information from one technology to another.

There are great potentials of using biosensors for in vivo and clinical applications due to their ability to provide rapid, sensitive, and specific detection of biomolecular interactions. Advances in biosensor design, including wearable and implantable devices, offer new avenues for real-time monitoring of biomarkers, disease progression, and therapeutic interventions. Key features, such as miniaturization, high sensitivity, and integration with digital health platforms, make biosensors particularly appealing for clinical diagnostics and monitoring. Despite these promising applications, significant challenges remain. In vivo environments are complex, with high variability and interference from nonspecific binding, molecular crowding, and fouling, all of which affect sensor accuracy and reliability. The presence of diverse biomolecules in clinical samples can lead to false positives and negatives, complicating the interpretation of sensor outputs. Additionally, the stability of biosensors under physiological conditions, including their longevity and resistance to biofouling, is critical for practical applications. This Review attempts to draw attention to the importance of the binding interaction profile and its kinetics in the development of more robust and reliable affinity biosensors, where more future studies in these aspects can bring existing innovative technologies closer to commercial stages.

## Vocabulary

binding kinetics: the rate of binding interaction between analyte and receptors in affinity biosensors
complex matrix: samples with target molecules and various none-target molecules that interfere with sensor
nonspecific binding: any interaction between nontarget molecules and receptors
secondary factors: any interfering factors affecting the binding interaction including receptor orientation, surface chemistry, external force fields, and environment of measurement
false-positive: detecting any interactions beyond target molecules in the sensor output signal
false-negative: any interference in binding interaction causing reduction of the sensor output signal;
